# Three independently deleted regions at chromosome arm 16q in human prostate cancer: allelic loss at 16q24.1–q24.2 is associated with aggressive behaviour of the disease, recurrent growth, poor differentiation of the tumour and poor prognosis for the patient

**DOI:** 10.1038/sj.bjc.6690025

**Published:** 1999-09-24

**Authors:** J P Elo, P Härkönen, A P Kyllönen, O Lukkarinen, P Vihko

**Affiliations:** Biocenter Oulu and WHO Collaborating Centre for Research on Reproductive Health, University of Oulu, Kajaanintie 50, Oulu, FIN-90220; Department of Pathology, University of Oulu, Kajaanintie 50, Oulu, FIN-90220; Department of Surgery, University of Oulu, Kajaanintie 50, Oulu, FIN-90220, Finland; Department of Biosciences, Division of Biochemistry, University of Helsinki, Helsinki, FIN-00014, Finland

**Keywords:** 16q, loss of heterozygosity, prostate cancer, HSD17B2, 17HSD type 2

## Abstract

Loss of heterozygosity at chromosome arm 16q is a frequent event in human prostate cancer. In this study, loss of heterozygosity at 16q was studied in 44 prostate cancer patients exhibiting various clinical features. Fifteen polymorphic polymerase chain reaction (PCR) markers were used to identify the separately deleted areas and the findings were compared with clinicopathological variables and 5-year survival of the patients. The results indicated that there are at least three independently deleted regions at 16q. Allelic losses at the central and distal areas were associated significantly with aggressive behaviour of the disease (16q24.1–q24.2, *P*< 0.01, and 16q24.3–qter, *P*< 0.05), and the central area of deletion was further significantly associated with poorly differentiated tumour cells (*P*< 0.05) and with recurrent (*P*< 0.01) growth of the tumour. During the follow-up period, 28% of the patients initially with M0 disease developed distant metastases. Of the patients showing allelic loss at 16q24.1–q24.2, distant metastasis were found in 45% during the 5-year follow-up period, and 31% of the patients showing loss at 16q21.1 also developed distant metastases. After the 5-year follow-up period, 14 (32%) of the patients remained alive, whereas 19 (43%) had died because of their prostate cancer. The overall survival rate of the patients showing allelic loss at 16q21.1 or 16q24.1–q24.2 was significantly lower than that of the patients with retained heterozygosity. © 1999 Cancer Research Campaign


*British Journal of Cancer* (1999) **79**(1), 156–160
© 1999 Cancer Research Campaign

### Three independently deleted regions at chromosome arm 16q in human prostate cancer: allelic loss at 16q24.1–q24.2 is associated with aggressive behaviour of the disease, recurrent growth, poor differentiation of the tumour and poor prognosis for the patient

**JP Elo[1], P Härkönen[1], AP Kyllönen[2], O Lukkarinen[3] and P Vihko[1,4]**

[1]Biocenter Oulu and WHO Collaborating Centre for Research on Reproductive Health, and Departments of [2]Pathology and [3]Surgery, University of Oulu, Kajaanintie 50, FIN-90220 Oulu, Finland; and [4]Department of Biosciences, Division of Biochemistry, University of Helsinki, FIN-00014 Helsinki, Finland

**Summary** Loss of heterozygosity at chromosome arm 16q is a frequent event in human prostate cancer. In this study, loss of heterozygosity at 16q was studied in 44 prostate cancer patients exhibiting various clinical features. Fifteen polymorphic polymerase chain reaction (PCR) markers were used to identify the separately deleted areas and the findings were compared with clinicopathological variables and 5-year survival of the patients. The results indicated that there are at least three independently deleted regions at 16q. Allelic losses at the central and distal areas were associated significantly with aggressive behaviour of the disease (16q24.1–q24.2, $P < 0.01$, and 16q24.3–qter, $P < 0.05$), and the central area of deletion was further significantly associated with poorly differentiated tumour cells ($P < 0.05$) and with recurrent ($P < 0.01$) growth of the tumour. During the follow-up period, 28% of the patients initially with M0 disease developed distant metastases. Of the patients showing allelic loss at 16q24.1–q24.2, distant metastasis were found in 45% during the 5-year follow-up period, and 31% of the patients showing loss at 16q21.1 also developed distant metastases. After the 5-year follow-up period, 14 (32%) of the patients remained alive, whereas 19 (43%) had died because of their prostate cancer. The overall survival rate of the patients showing allelic loss at 16q21.1 or 16q24.1–q24.2 was significantly lower than that of the patients with retained heterozygosity.

**Keywords:** 16q; loss of heterozygosity; prostate cancer; HSD17B2; 17HSD type 2

Prostate cancer is one of the most common cancers among men in many Western countries, including Finland. However, the molecular genetic events associated with the development and progression of the disease are poorly known. According to the multistep model of carcinogenesis, several genetic aberrations take place in prostate cancer, but only a few genes potentially involved in prostatic carcinogenesis have been identified.

It has been suggested that genetic alterations take place in several chromosomal regions in prostate cancer and, in comparative genomic hybridization (CGH) studies, the long arm of chromosome 16 is among those showing the most frequent loss (Joos et al, 1995; Visakorpi et al, 1995; Cher et al, 1996). Losses at chromosome 16 have been reported to occur almost exclusively in the long arm (q) (Bergerheim et al, 1991; Visakorpi et al, 1995; Cher et al, 1996). Both primary and metastatic tumours have been found to show allelic loss at 16q, and the occurrence of loss of heterozygosity (LOH) has also been associated with clinicopathological variables such as aggressive and metastatic behaviour of the disease and poor differentiation of the tumour (Carter et al, 1990; Suzuki et al, 1996; Elo et al, 1997; Latil et al, 1997). The most common area of deletion has been suggested to be distal in 16q, but the most recent results have indicated evidence of loss of several independent regions in 16q (Suzuki et al, 1996; Elo et al, 1997; Latil et al, 1997).

Particular interest in losses at 16q has been paid to the region 16q21.1, where a potential tumour-suppressor gene, that for E-cadherin, is located. Decreased E-cadherin expression has been associated with poor prognosis of prostate cancer (Umbas et al, 1994). Recently, the regions of loss at chromosome arm 16q have been narrowed down. It has been suggested in several reports that losses at 16q are concentrated in three independent regions (Suzuki et al, 1996; Latil et al, 1997). The proximal area of loss is suggested to be located somewhere at 16q21.1 and the distal area of loss has been suggested to lie at 16q24.3. The central region of loss has been reported to be at 16q23.2 (Latil et al, 1997), but it has also been reported to be located at 16q23.2–q24.1 (Suzuki et al, 1996). Our recent data indicate that a 7.6-cM central area of loss is located at 16q24.1–q24.2, between markers D16S504 and D16S422. In addition, LOH at 16q24.1–q24.2 (HSD17B2 and D16S422) was found to be the most frequent area of deletion, and it was significantly correlated with aggressive and metastatic behaviour of the disease and also with poorly differentiated tumours (Elo et al, 1997).

There are several candidate genes putatively involved in prostatic carcinogenesis located distally at 16q. The 17HSD type 2 gene (*HSD17B2*), located at 16q24.1–q24.2, encodes the 17β-hydroxysteroid dehydrogenase (17HSD) type 2 isoenzyme, which

*Received 10 February 1998*
*Revised 11 May 1998*
*Accepted 19 May 1998*

*Correspondence to:* P Vihko, Biocenter Oulu and WHOCCR, University of Oulu, Kajaanintie 50, FIN-90220 Oulu, Finland has been reported to inactivate active androgens in prostatic epithelial cells and is, thus, suggested to protect them from excessive androgen action (Délos et al, 1995; Durocher et al, 1995; Elo et al, 1996). The genes for three cell adhesion-regulating molecules are also reported to be located at 16q24. The cell adhesion regulator gene is located at 16q24 (Pullmann and Bodmer, 1992). Decrease of its expression could be involved in decreased tumour invasion suppression. The genes of two members of the cadherin superfamily, *M-cadherin* and *H-cadherin*, are located at 16q24 (Kaupman et al, 1992; Lee, 1996) and the expression of H-cadherin has been reported to be decreased in breast tumours (Lee, 1996). C-*myc* promoter-binding protein, whose gene is another possible target, is located at 16q24 and it is a negative regulator of c-*myc* oncogene expression (Ray and Miller, 1991).

In the present study, LOH at chromosome arm 16q was studied in 44 prostatic cancer patients exhibiting various clinical features. Fifteen polymorphic polymerase chain reaction (PCR) markers were used to identify the separately deleted areas, and the findings were compared with clinicopathological variables and the 5-year survival rate of the patients. Exons 1–3 and 6–7 of the gene *HSD17B2*, coding for active 17HSD type 2 enzyme, were sequenced using tissues from 20 patients showing LOH at 16q24.1–q24.2.

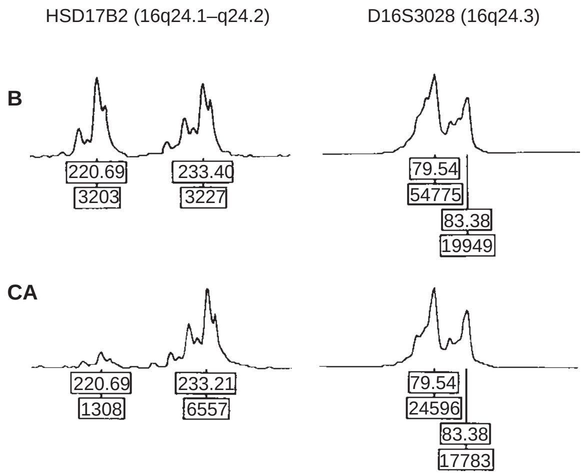

**Figure 1** An example of a prostate cancer specimen showing loss of heterozygosity at 16q24.1–q24.2 (HSD17B2) and retained heterozygosity at 16q24.3 (D16S3028). Fragment size is indicated in the upper box and the area of the peak in the lower box

### MATERIALS AND METHODS

### Subjects and specimens

Allelic losses at chromosome arm 16q were studied in tissues from 44 prostate cancer patients undergoing radical prostatectomy or transurethral resection of the prostate. The patients were classified according to the TNM classification system (T1M0, three patients; T2M0, six patients; T3M0, 13 patients; T4M0, nine patients; T3M1, eight patients; T4M1, five patients) (Chisholm, 1988). Digital rectal examination, transrectal ultrasonography, bimanual palpation and cystoscopy were used when determining the T-stage. The M-stage was determined in all the patients by bone scanning, thorax radiograph and ultrasound examination of the abdomen; to determine the N-stage, staging pelvic lymphadenectomy was carried out only when radical operation was performed. Twenty of the patients were operated upon because of recurrent disease after endocrine therapy and the remaining twenty-four patients in the study had primary tumours. Tumours without local progression or metastases and all grade 1 tumours were considered clinically to be non-aggressive (26 patients). The disease of 18 of the patients was considered clinically aggressive.

The specimens were histologically confirmed to contain at least 60% malignant cells. After dissecting the specimens from paraffin blocks, the DNA was extracted using a standard protocol (Wright and Manos, 1990). For controls, DNA obtained from benign prostatic tissue of the same patient was used.

### PCR and fragment analysis

Fifteen microsatellite markers (D16S408, D16S514, D16S503, D16S512, D16S515, D16S516, D16S504, D16S511, HSD17B2, D16S422, D16S3061, D16S520, D16S476, D16S3028 and D16S413) spanning chromosomal arm 16q and their corresponding PCR primers were chosen from the Genethon comprehensive genetic map (Dib et al, 1996) or from the Genomic Data Base (http://gdbwww.gdb.org/). The 25-µl PCR mixtures contained 100 ng template DNA, 50 pmol each of two primers (one of them fluorescently labelled; Pharmacia LKB Biotechnology), 200 µmol of each deoxy-NTP (Pharmacia LKB Biotechnology), Reaction Buffer IV (Advanced Biotechnologies), 1 mM magnesium chloride (Advanced Biotechnologies) and 0.25 U of Red Hot DNA-polymerase (Advanced Biotechnologies). According to the hot start protocol, the reaction mixtures were first denatured at 95°C for 5 min, after which the DNA polymerase was added. Thirty-five PCR cycles were then carried out (GeneAmp 9600 thermal cycler, Perkin Elmer Cetus) using the following steps: denaturing at 95°C for 1 min, annealing at 55–65°C (optimized separately for each primer pair) for 1 min, and amplification at 72°C for 2 min. Lastly, a 7-min final extension at 72°C was carried out. To determine the sizes of the alleles and the loss of heterozygosity, the fluorescent PCR products were loaded on a 5% Long Ranger gel (FMC BioProducts) in the presence of Prism Genescan-500 Tamra allelic size markers (Applied Biosystems) and run for 4 h in an ABI 377 sequencer utilizing GeneScan software (Applied Biosystems). The data obtained were analysed by using Genotyper software (Applied Biosystems). Analysis of LOH was carried out by calculating the ratio of the constituent alleles, as reported previously (Canzian et al, 1996; Elo et al, 1997). When one of the alleles was decreased by at least 40%, the specimen was considered to show LOH. An example of representative results obtained is shown in Figure 1.

### Direct sequencing

In twenty specimens showing LOH at 16q24.1–q24.2, the exons of *HSD17B2* coding for the active enzyme were sequenced. The primers used for amplication of exons 1–3 and 6–7 encoding 17HSD type 2 were designed on the basis of the nucleotide sequence of *HSD17B2* (Labrie et al, 1995). PCR amplification was performed as described for LOH analysis above, except that the magnesium chloride concentration was increased to 1.5 mmol $l^{-1}$, and the dNTP and primer concentrations were decreased to 0.08 mmol $l^{-1}$ and 20 pmol $l^{-1}$ respectively. The PCR products were purified from excess primers and nucleotides by using a single-step ExoI/SAP method (Werle et al, 1994).

© Cancer Research Campaign 1999

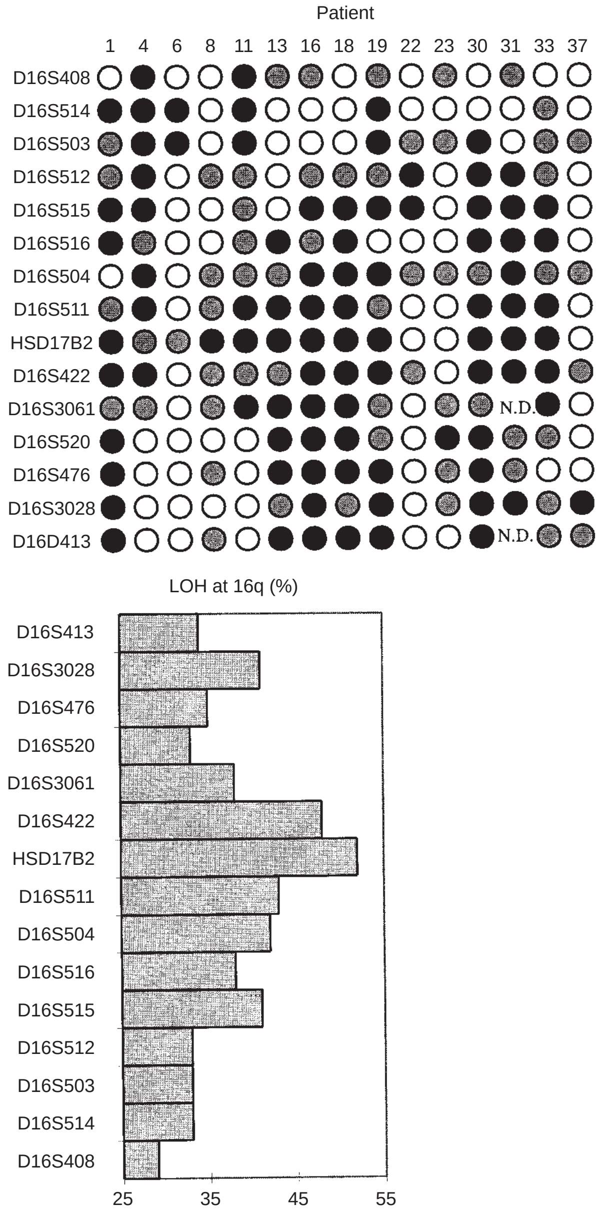

**Figure 2** (**A**) Deletion map of the tumours showing partial LOH at chromosome arm 16q. (○) Retention of heterozygosity; (●) loss of heterozygosity; (●) homozygote. The data indicate that there are at least three independently deleted regions at 16q. The proximal area of common loss is at 16q21.1 (D16S514–D16S503), the central area of deletion is at 16q24.1–q24.2 (D16S504–D16S422) and the distal region is at 16q24.3–qter (D16S3028–qter). The central region is further suggested to be the most commonly deleted independent area of deletion. (**B**) Percentage of prostate cancer specimens showing allelic loss of the markers studied. The most commonly deleted markers were HSD17B2 (52%) and D16S422 (48%), located at 16q24.1–q24.2

Cycle sequencing reactions using an Abi Prism dRhodamine Terminator Cycle Sequencing Ready Reaction Kit were carried out in a total reaction volume of 20 μl, as suggested by the manufacturer (Applied Biosystems). The extension products were purified by using an ethanol/sodium acetate precipitation procedure and the samples were prepared according to the instructions of the manufacturer (Applied Biosystems). In gel electrophoresis, the samples were loaded on a 5% Long Ranger gel (FMC Bio Products) and run in the Abi Prism 377 DNA Sequencer (Perkin Elmer Cetus). The results obtained were analysed using DNA Sequencing Analysis 2.1.1 software (Applied Biosystems).

### Statistical analysis

Two-tailed Fisher's exact tests (SPSS for Windows, SPSS) were used to analyse the associations between loss of heterozygosity and the clinicopathological parameters. In addition, backward step-wise logistic regression analysis (SPSS for Windows, SPSS) was used in testing the associations, and the survival analyses were carried out by using the Kaplan–Meier test (SPSS for Windows, SPSS). A $P$-value of < 0.05 was considered as significant.

### RESULTS

Regions of common allelic loss were studied in 44 prostatic cancer specimens and in corresponding benign specimens, using 15 microsatellite markers. Twenty-two of the specimens (50%) showed LOH at chromosome arm 16q, 15 of the specimens (34%) showed partial loss at 16q, whereas seven of the specimens (16%) showed complete loss of the segments studied. The data indicated that there are at least three independently deleted regions, as reported previously. In the present study, the most proximally located 1.8-cM area of loss was at 16q21.1 (region A), between markers D16S514 and D16S503; the central 7.6-cM area of deletion was at 16q24.1–q24.2 (region B) between markers D16S504 and D16S422; and the most distal area of common deletion was located at 16q24.3–qter (region C), distal to marker D16S3028 (Figure 2A and B).

Of the three independently deleted areas, region A showed LOH in 14 (32%), region B in 19 (43%) and region C in 16 (36%) of the specimens studied. The most commonly deleted individual markers, D16S422 (48%, 15/31) and HSD17B2 (52%, 16/31), were located within region B. Among the cancer death cases, LOH at 16q was found in 63% (12/19) of the specimens. The 13 metastatic disease showed LOH at the three independent regions in 31% (region A) to 62% (region B) of the specimens. Similarly, the frequency of losses at the separate regions in aggressive diseases varied from 43% (region A) to 67% (region B) of the specimens.

The frequency of losses at the independently deleted regions was further compared with the clinicopathological variables. The data indicated that losses at regions A, B and C were significantly associated with aggressive behaviour of the disease (A, $P < 0.05$; B, $P < 0.01$; C, $P < 0.05$). In addition, losses at region B were significantly associated with poorly differentiated tumour cells ($P < 0.05$) and with recurrent ($P < 0.01$) growth of the tumour. Step-wise logistic regression analysis showed that aggressive behaviour of the disease and recurrent growth were correlated significantly with the appearance of LOH at region B ($P < 0.01$). Recurrent growth was associated with an approximately sixfold increase in the occurrence of LOH, and aggressive disease was associated with a sevenfold increase in the appearance of LOH.

During the 5-year follow-up period, 28% of the patients initially with M0 disease developed distant metastases. Of the M0 patients showing LOH at region B, 45% developed metastases, and metastases were found in 28% of the patients showing LOH at region A. Interestingly, only 17% and 19%, respectively, of the patients with retained heterozygosity at these two regions developed metastatic disease. After the 5-year follow-up period, 14 (32%) of the patients remained alive, whereas 19 (43%) had died as a result of their prostate cancer and 11 (25%) had died as a result of another

*© Cancer Research Campaign 1999*

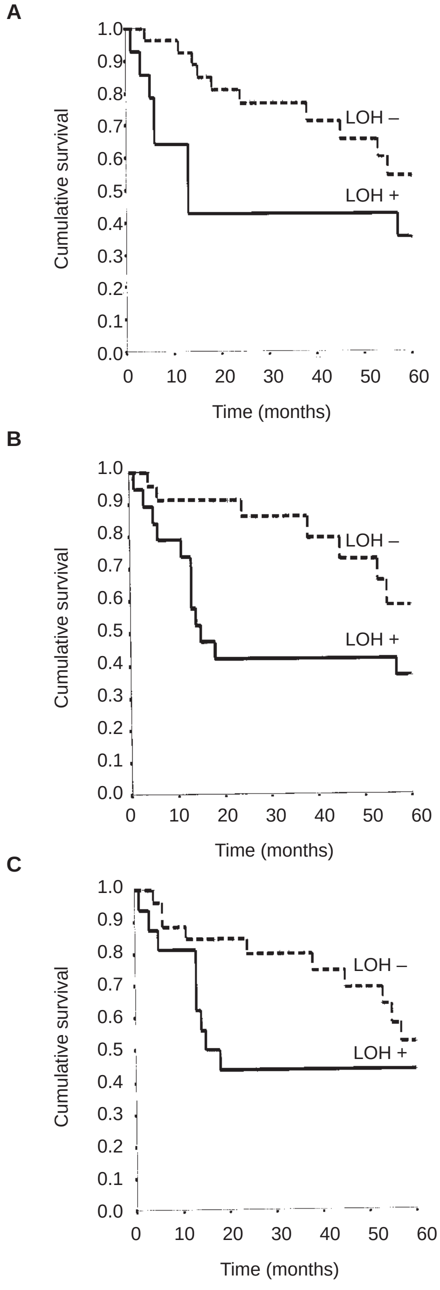

**Figure 3** Kaplan–Meier overall survival rates of the 44 prostate cancer patients exhibiting various clinical features, related to LOH detected. (**A**) Region A (16q21.1; *P* = 0.014, Breslow test), (**B**) region B (16q24.1–q24.2; *P* = 0.011, Breslow test) and (**C**) region C (16q24.3–qter; *P* = 0.072, Breslow test)

cause. The overall survival rate of the patients showing allelic loss at regions A and B was significantly lower than that of the patients with retained heterozygosity (Figure 3). In addition, the survival rate of the patients with recurrent disease and showing allelic loss at region B was significantly lower ($P = 0.041$; Breslow test) than that of the patients with recurrent disease, but retained heterozygosity. Sequencing analysis of the coding regions of *HSD17B2* revealed no amino acid-changing point mutations.

### DISCUSSION

Deletion mapping of the long arm of chromosome 16 indicated loss at three independent regions at least, as reported previously in two separate studies (Suzuki et al, 1996; Latil et al, 1997). The proximal area of deletion in the present study was located at 16q21.1 (region A), whereas the central area was located at 16q24.1–q24.2 (region B) and the distal area was located at 16q24.3–qter (region C). There is consistency as regards the location of frequent loss at 16q21.1 between studies, but results concerning the central area are more controversial. Our previous data indicated a common area of loss located centrally at 16q24.1–q24.2, between markers D16S504 and D16S422. The central area bracketed by Latil et al (1997) was located at 16q23.2, and Suzuki et al (1996) located the central area at 16q23.2–q24.1. Data from previous studies agree as regards the location of the third and most distal area of deletion as being from 16q24.3 to qter.

The deletion pattern observed in 16q in the present study indicates that the most commonly deleted individual markers (D16S422 and HSD17B2) were located within region B. The same markers were also the most commonly deleted in our previous study, and, in addition, loss at the region was associated with aggressive features of the disease (Elo et al, 1997). Although the number of patients studied was relatively small, the findings were compared with the clinicopathological features. The association between loss at region B and clinically aggressive features of the disease, suggested in our previous study, was confirmed. Loss at region B was found to be significantly correlated with poor differentiation of the tumour cells, aggressive behaviour of the disease and recurrent growth of the tumour at the time of obtaining the specimen. Interestingly, losses at regions A and C were associated only with aggressive behaviour of the disease.

The frequent occurrence of common areas of loss at 16q is of particular interest because of the clinical associations and the identification of several genes putatively involved in prostatic carcinogenesis (Carter et al, 1990; Visakorpi et al, 1995; Suzuki et al, 1996; Elo et al, 1997; Latil et al, 1997). Loss at 16q24.1–q24.2 separately from the distal region at 16q24.3–qter further supports the idea of the existence of at least two different genes putatively involved in prostatic carcinogenesis located distally at chromosome arm 16q. Despite the identification of some of the genes located at frequently deleted regions, none of the candidate genes have been confirmed to be the main target of the losses observed. The genes for at least two different adhesion molecules are located at 16q24. M-cadherin, whose gene is located at the central region of loss, is a member of the superfamily of cell adhesion molecules. Interestingly, the gene for another cell adhesion molecule, H-cadherin, the decreased expression of which has been reported in breast cancer tissue, is located at 16q24 (Lee, 1996). The cell adhesion regulator gene is located at 16q24.3, within the distal region of common loss. In addition to the cell adhesion molecule genes, that for c-*myc* promoter-binding protein, which regulates the expression of the c-*myc* oncogene, is also located at 16q24 (Ray and Miller, 1991). The gene *HSD17B2*, located at 16q24.1–q24.2, is also a gene of interest. The gene encodes the enzyme 17HSD type 2, which inactivates the active androgens testosterone and 5α-dihydrotestosterone into their less active metabolites androstenedione and 5α-androstenedione respectively. Sequencing of the exons coding for active 17HSD type 2 showed no amino acid-changing point mutations, but it is still possible that mutations affecting the transcriptional activity of the gene are

© Cancer Research Campaign 1999

located at the promoter area not analysed in the present study. In addition, the possible methylation sites of *HSD17B2* are not known. A decrease in the expression of 17HSD type 2 could lead to increases in the concentrations of active androgens in the prostate and, thus, result in an increase in proliferative pressure as a consequence of the presence of active androgens in the prostatic epithelium. The expression of 17HSD type 2 mRNA is reported to be lower in prostate cancer tissues, when compared with that in hyperplastic prostate tissue (Elo et al, 1996). In addition, 17HSD activity has been reported to be lower in a group of patients with poor responses to androgen ablation therapy (Brendler et al, 1984). It has been suggested that allele loss could lead to decreased gene expression as a result of the 'genetic dosage' effect (Vogelstein and Kinzler, 1992; Isaacs, 1994). The frequent LOH detected at 16q24.1–q24.2 could be one explanation for the decreased 17HSD mRNA expression and 17HSD activity detected in prostate cancer specimens (Brendler et al, 1984; Klein et al, 1991; Elo et al, 1996).

The present data further confirm the existence of at least three independently deleted regions at 16q. It is, therefore, suggested that several important genes regarding prostate cancer are located at chromosome arm 16q and in particular at 16q24.1–q24.2.

### ACKNOWLEDGEMENTS

The authors would like to thank Ms Mirja Mäkeläinen for her expert technical assistance. The work was supported by the Research Council of Health of the Academy of Finland (project nos. 3314 and 30099) and the Technology Development Center of Finland (TEKES, project no. 4476). The WHO Collaborating Centre for Research on Reproductive Health is supported by the Ministries of Education, Social Affairs and Health, and Foreign Affairs, Finland.

### REFERENCES

Bergerheim US, Kunimi K, Collins VP and Ekman P (1991) Deletion mapping of chromosomes 8, 10, and 16 in human prostatic carcinoma. *Genes Chroms Cancer* **3**: 215–220

Brendler CB, Isaacs JT, Follansbee AL and Walsh PC (1984) The use of multiple variables to predict response to endocrine therapy in carcinoma of the prostate: a preliminary report. *J Urol* **131**: 694–700

Canzian F, Salovaara R, Hemminki A, Kristo P, Chadwick RB, Aaltonen LA and de la Chapelle A (1996) Semiautomated assessment of loss of heterozygosity and replication error in tumors. *Cancer Res* **56**: 3331–3337

Carter BS, Ewing CM, Ward WS, Treiger BF, Aalders TW, Schalken JA, Epstein JI and Isaacs WB (1990) Allelic loss of chromosomes 16q and 10q in human prostate cancer. *Proc Natl Acad Sci USA* **87**: 8751–8755

Cher ML, Bova GS, Moore DH, Small EJ, Carroll PR, Pin SS, Epstein JI, Isaacs WB and Jensen RH (1996) Genetic alterations in untreated metastases and androgen-independent prostate cancer detected by comparative genomic hybridization and allelotyping. *Cancer Res* **56**: 3091–3102

Chisholm GD (1988) TNM classification of urologic tumours in 1988. *Br J Urol* **62**: 501

Délos S, Carsol J-L, Ghazarossian E, Raynaud J-P and Martin P (1995) Testosterone metabolism in primary cultures of human prostate epithelial cells and fibroblasts. *J Steroid Biochem Mol Biol* **55**: 375–383

Dib C, Faure S, Fizames C, Samson D, Drout N, Vignal A, Millasseau P, Marc S, Hazan J, Seboun E, Lathrop M, Gyapay G, Morrissette J and Weissenbach J (1996) A comprehensive genetic map of the human genome based on 5,264 microsatellites. *Nature* **380**: 152–154

Durocher F, Morrisette J, Labrie Y, Labrie F and Simard J (1995) Mapping of the HSD17B2 gene encoding type II 17β-hydroxysteroid dehydrogenase close to D16S422 on chromosome 16q24.1–q24.2. *Genomics* **25**: 724–726

Elo JP, Akinola LA, Poutanen M, Vihko P, Kyllönen A-P, Lukkarinen O and Vihko R (1996) Characterization of 17β-hydroxysteroid dehydrogenase isoenzyme expression in benign and malignant human prostate. *Int J Cancer* **66**: 37–41

Elo JP, Härkönen P, Kyllönen A-P, Lukkarinen O, Poutanen M, Vihko R and Vihko P (1997) Loss of heterozygosity at 16q24.1–q24.2 is significantly associated with metastatic and aggressive behavior of prostate cancer. *Cancer Res* **57**: 3356–3359

Isaacs JT (1994) Role of androgens in prostatic cancer. *Vitam Horm* **49**: 433–502

Joos S, Bergerheim USR, Pan Y, Matsuyama H, Bentz M, du Manoir S and Lichter P (1995) Mapping of chromosomal gains and losses in prostate cancer by genomic hybridization. *Genes Chromosomes Cancer* **14**: 267–276

Kaupmann K, Becker-Follmann J, Scherer G, Jockusch H and Starzinski-Powitz A (1992) The gene for cell adhesion molecule M-cadherin maps to mouse chromosome 8 and human chromosome 16q24.2–qter and is near the E-cadherin (Uvomorulin) locus in both species. *Genomics* **14**: 488–490

Klein H, Bressel M, Kastendieck H and Voigt K-D (1991) Biochemical endocrinology of prostate cancer. In *Endocrine Dependent Tumors*. Voigt K-D, Knabbe K (eds), pp. 131–163. Raven Press: New York

Labrie Y, Durocher F, Lachance Y, Turgeon C, Simard J, Labrie C and Labrie F (1995) The human type 2 17β hydroxysteroid dehydrogenase gene encodes two alternatively spliced mRNA species. *DNA Cell Biol* **14**: 849–861

Latil A, Cussenot O, Fournier G, Driouch K and Lidereau R (1997) Loss of heterozygosity at chromosome 16q in prostate adenocarcinoma: identification of three independent regions. *Cancer Res* **57**: 1058–1062

Lee SW (1996) H-cadherin, a novel cadherin with growth inhibitory functions and diminished expression in human breast cancer. *Nature Med* **2**: 776–782

Pullman WE and Bodmer WF (1992) Cloning and characterization of a gene that regulates cell adhesion. *Nature* **356**: 529–532

Ray R and Miller DM (1991) Cloning and characterization of a human c-myc promoter-binding protein. *Mol Cell Biol* **11**: 2154–2161

Suzuki H, Komiya A, Emi M, Kuramochi H, Shiraishi T, Yatani R and Shimazaki J (1996) Three distinct commonly deleted regions of chromosome arm 16q in human primary and metastatic prostate cancer. *Genes Chroms Cancer* **17**: 225–233

Umbas R, Isaacs WB, Bringuier PP, Schaafsma HE, Karthaus HFM, Oosterhof GON, Debruyne FMJ and Schalken JA (1994) Decreased E-cadherin expression is associated with poor prognosis in patients with prostate cancer. *Cancer Res* **54**: 3929–3933

Visakorpi T, Kallioniemi AH, Syvänen A-C, Hyytinen ER, Karhu R, Tammela T, Isola JJ and Kallioniemi OP (1995) Genetic changes in primary and recurrent prostate cancer by comparative genomic hybridization. *Cancer Res* **55**: 342–347

Vogelstein B and Kinzler KW (1992) p53 Function and dysfunction. *Cell* **70**: 523–526

Werle E, Schneider C, Völker M and Fiehn W (1994) Convenient single-step, one tube purification of PCR products for direct sequencing. *Nucleic Acids Res* **22**: 4354–4355

Wright DK and Manos MM (1990) Sample preparation from paraffin-embedded tissues. In *PCR Protocols*: *A Guide to Methods and Applications*. Innis MA, Gelfand JJ and White TJ (eds), pp. 153–158. Academic Press: San Diego

 *© Cancer Research Campaign 1999*
